# Heat‐shock protein 27 (HSP27, HSPB1) is synthetic lethal to cells with oncogenic activation of MET, EGFR and BRAF

**DOI:** 10.1002/1878-0261.12042

**Published:** 2017-05-08

**Authors:** John D. Konda, Martina Olivero, Daniele Musiani, Simona Lamba, Maria F. Di Renzo

**Affiliations:** ^1^ Department of Oncology University of Torino Italy; ^2^ Candiolo Cancer Institute FPO‐IRCCS Italy; ^3^ Present address: Department of Experimental Oncology European Institute of Oncology Milan Italy

**Keywords:** oncogenes, small heat‐shock proteins, target therapy

## Abstract

The small heat‐shock protein of 27 kDa (HSP27) is highly expressed in many cancers and is associated with aggressive tumour behaviour, metastasis, poor prognosis and resistance to chemotherapy. We aimed at assessing the role of HSP27 in modulating responses to target therapies. We selected several oncogene‐addicted cancer cell lines, which undergo either cell cycle blockade or cell death in response to agents that target the specific oncogene. Surprisingly, HSP27 suppression alone resulted in the apoptotic death of MET‐addicted EBC‐1 lung cancer cells, epidermal growth factor receptor (EGFR)‐addicted colorectal carcinoma (CRC) DiFi cells and BRAF‐addicted CRC COLO205 and OXCO‐1 and melanoma COLO741 cells, all of which also undergo death when treated with the specific targeted agent. In other cell lines, such as MET‐addicted gastric carcinoma MKN45 and EGFR‐addicted CRC SW48 lines, where oncogene inhibition only blocked proliferation, HSP27 knockdown made targeted agents switch from cytostatic to cytotoxic activity. Mechanistically, the more the cells were susceptible to HSP27 suppression, the more they were primed for death, as demonstrated by increased levels of mitochondrial outer membrane permeabilization. Priming for death was accompanied by the increase in pro‐apoptotic proteins of the BCL2 family and of active caspase‐3 and lamin B. Together, these data suggest that oncogene‐addicted cells require HSP27 for survival and that HSP27 might interfere with the effectiveness of targeted agents.

**Abbreviations**
EGFR: epidermal growth factor receptor
HSP27: heat‐shock protein of 27 kDa
MOMP: mitochondrial outer membrane permeabilization

## 1 Introduction

The most successful current paradigms in targeted therapy consist of directly inhibiting mutated or amplified oncogenes, to which the cancer cells, but not the normal cell counterpart, are ‘addicted’. Depriving the addicted cancer cell of this signal leads to cell death or at least growth arrest. In human cancer treatment, the most convincing evidence came out, supporting the concept of ‘oncogene addiction’, as there are examples of the therapeutic efficacy of antibodies or drugs targeting specific oncogenes.

Among known oncogenes, those encoding kinases account for a significant number of molecular targets exploited today in the clinic. Their targeting agents might be small‐molecule inhibitors, which block kinase enzymatic activity or signalling (for a review, see Gross *et al*., 2015). For example, the inhibition of the activated *BRAF* oncogene with small molecules represents an important therapeutic approach. The V600 BRAF mutation is the most common in melanoma, and mutation‐specific inhibitors are effectively used to treat melanoma and some of the nonmelanoma cancers with the same mutation (Hyman *et al*., 2015). When oncogenic kinases are receptors, monoclonal antibodies can be used that bind to the extracellular region of the receptor and interfere at varying levels with its activity (for a review, see Weiner, 2015). Monoclonal antibodies interfering with the signalling of the hepatocyte growth factor receptor encoded by the *MET* oncogene and of the epidermal growth factor receptor (EGFR) encoded by the *HER1* gene have received approval for the treatment of various types of cancers. Drugging the *RAS* oncogene, a small GTPase, came out to be by far more difficult, although of paramount importance, *RAS* being among the most common oncogenic drivers in human malignancies. Activating *RAS* mutations are associated with approximately 30% of human cancers that are frequently resistant to standard therapies. The addiction of these cancers to *RAS* activation has been studied. A better understanding of *RAS* structure, biochemistry, processing and signalling will open new possibilities to overcome *RAS*‐driven tumours. Recently, efforts to develop direct inhibitors of RAS have gathered new momentum (for reviews, see Cox *et al*., 2014; Ostrem and Shokat, 2016; Singh *et al*., 2015).

The small heat‐shock proteins (sHSPs) are molecular chaperones that play a role in development, stress responses and diseases, including cancer (Lanneau *et al*., 2010; Zoubeidi and Gleave, 2012). The small heat‐shock protein of 27 kDa (HSP27) encoded by the *HSPB1* gene shows anti‐aggregation property, as it participates in sequestering damaged proteins (Garrido *et al*., 2012) and is involved in the proteasomal degradation of proteins under stress conditions (Lanneau *et al*., 2010). HSP27 also exerts important anti‐apoptotic function by binding apoptotic proteins (for a review, see Acunzo *et al*., 2012). Clinically, HSP27 is highly expressed in many cancers including breast (Conroy *et al*., 1998), ovarian (Arts *et al*., 1999), prostate (Cornford *et al*., 2000) and others (Bruey *et al*., 2000) and is associated with aggressive tumour behaviour, metastasis and poor prognosis. HSP27 increases during the early phase of stem cell differentiation (Mehlen *et al*., 1997), and thus, it might play a role in sustaining cancer stem cell growth and survival. It is noteworthy that HSP27 suppression alone resulted in the death of prostate (Zoubeidi *et al*., 2007) and pancreatic carcinoma cells (Baylot *et al*., 2011). Moreover, HSP27 inhibition increases the efficacy of the chemotherapeutic agents such as gemcitabine (Baylot *et al*., 2011), paclitaxel (Andrieu *et al*., 2010; Pavlíková *et al*., 2015) and doxorubicin (Díaz‐Chávez *et al*., 2013). However, in pancreatic cancer a recent conflicting report associates an increased phosphorylation of HSP27 with better prognosis (Okuno *et al*., 2016). Hence, HSP27 might play important roles in cancer onset and progression and in its response to treatment. We (Musiani *et al*., 2014) and others (Lelj‐Garolla *et al*., 2015) have shown that targeted agents might upregulate HSP27 expression weakening the efficacy of inhibitors.

Here, we show that HSP27 might impair the efficacy of targeted agents in many contexts of oncogene addiction through its role in protecting cells from mitochondrial priming.

## 2 Materials and methods

### 2.1 Cell lines and reagents

Most cell lines used were purchased from the American Type Culture Collection (ATCC) after 2011, unless otherwise specified. All colorectal carcinoma (CRC) cell lines used were kindly provided by Professor Alberto Bardelli, Department of Oncology, University of Torino, Italy. DiFi and OXCO‐1 cell lines were a generous gift from Dr J. Baselga in November 2004 (Oncology Department of Vall d'Hebron University Hospital, Barcelona, Spain) and Dr V. Cerundolo in March 2010 (Weatherall Institute of Molecular Medicine, University of Oxford, UK), respectively. All cell lines were cultured as suggested by the provider. JNJ‐38877605 was obtained from Janssen Pharmaceutical (Johnson & Johnson, New Brunswick, NJ, USA). Cetuximab (CTX) was purchased from Merck KGaA (Darmstadt, Germany). PLX4720 was obtained from Selleck Chemicals.

### 2.2 Quantitative PCR

Quantitative PCR was carried out as described previously (Bardella *et al*., 2007). Total cellular RNA was isolated using the SV Total RNA Isolation kit (Promega, Fitchburg, WI, USA). To quantify the expression levels of HSP27‐encoding gene, equal amounts of cDNA were synthesized using the Moloney murine leukaemia reverse transcriptase (Promega) and mixed with SsoFast EvaGreen Supermix (Bio‐Rad, Hercules, CA, USA) and 300 μm of each of the respective forward and reverse primers. Quantitative real‐time PCR was performed on a MyiQ thermal cycler (Bio‐Rad). Target gene expression was evaluated using a relative quantification approach, with POLR2A (GenBank accession no. NM000937.4) as an internal reference. Primer sets used are as follows: POLR2A: forward TG‐CAAGGGCAAAAACATATGC, reverse AGCTCTAGGCCA‐GAACGCC; HSP27: forward GCGTGTCCCTGGATGTCAAC, reverse TGTATTTCCGCGTGAAGCAC. PCR cycling conditions were as follows: 30 s at 95 °C 30, 5 s at 95 °C plus 15 s at 60 °C (40 cycles), 30 s at 95 °C, and 10 s at 65 °C plus 10 s at 0.5 °C (60 cycles: melting curve).

### 2.3 Western blot analysis

Western blot analysis was carried out as described previously (Musiani *et al*., 2014) using the following antibodies: mouse monoclonal anti‐vinculin from Sigma‐Aldrich (St. Louis, MO, USA); mouse monoclonal anti‐HSP27 and mouse monoclonal anti‐ERK and anti‐P‐ERK (Thr202/Tyr204) from Cell Signaling Technology (Beverly, MA, USA); and mouse monoclonal anti‐KRAS from Santa Cruz Biotechnology (Santa Cruz, CA, USA).

### 2.4 RNA interference

Transient silencing of HSP27 by RNA interference was performed using On‐TargetPlus SmartPool (Dharmacon, Lafayette, CO, USA). In each experiment, the On‐TargetPlus nontargeting pool (Dharmacon) was used as the negative control. The cell lines were plated at 30–40% confluency and transfected with the indicated siRNA pools (100 nm) using oligofectamine (Invitrogen, Eugene, OR, USA), according to the manufacturer's instructions. Stable silencing of HSP27 was achieved using specific shRNAs transduced into the cells by means of lentiviral vectors. HSP27‐specific shRNAs (TRCN0000008753 and TRCN0000342857) were obtained from the Sigma‐Aldrich TRC lentiviral shRNA library (Sigma‐Aldrich). Transient silencing of KRAS was achieved as described (Lamba *et al*., 2014).

### 2.5 Cell transduction with lentiviral vectors

Cells were transduced using second‐generation lentiviral vectors, whose stocks were produced by transient transfection of 293T cells with the packaging plasmid pCMV‐DR8.74, the envelope plasmid pMD2G‐V respective transfer gene‐carrying vector. Serial dilutions of freshly harvested conditioned medium were used to infect cells in a six‐well plate in the presence of polybrene (8 μg·mL^−1^).

### 2.6 Apoptosis and viability assays

Apoptosis was measured as staining with APC‐conjugated annexin V (Bender MedSystems, Burlingame, CA, USA) and DAPI (Invitrogen), in accordance with the manufacturer's instructions. The samples were analysed on CyAn‐ADP flow cytometer (Dako, Carpinteria, CA, USA). Data acquisition was performed using summit software (Dako). To assess growth inhibition, a viability assay was used, seeding cells at density optimized for 96‐well plates. The cells were cultured in the presence of drugs or vehicle and subjected to the CellTiter‐Glo luminescent cell viability assay (Promega) in at least triplicate samples according to the manufacturer's specifications. The results are an average of three independent assays. Apoptosis and growth inhibition shown were always measured in parallel experiments.

### 2.7 Immunoassay of apoptotic proteins

Human pro‐ and anti‐apoptotic proteins were measured using the Bio‐Plex^®^ Multiplex Immunoassays (Bio‐Rad Laboratories, Hercules, CA, USA) based on the Luminex technology (Luminex, Austin, TX, USA) and carried out at Bioclarma srl, Torino, Italy. Briefly, the assay combines the principle of a sandwich immunoassay with fluorescent bead‐based technology, thus allowing individual and multiplex analysis of different analytes in a single test tube (Vignali, 2000). Capture antibodies directed against the desired biomarker are covalently coupled to fluorescently dyed magnetic microspheres, each with a distinct colour code or spectral address to allow identification of individual proteins within a multiplex suspension. A biotinylated detection antibody is added to create a sandwich complex. The final detection complex is formed with the addition of streptavidin–phycoerythrin (SA‐PE) conjugate and measured with the Bio‐Plex 100 System array reader (Bio‐Rad Laboratories), which identifies and quantifies each specific reaction based on bead colour and fluorescent signal intensity. Data were processed using bio‐plex manager software (version 6.1) (Bio‐Rad Laboratories) using five‐parametric curve fitting and converted in ng·mL^−1^.

### 2.8 Mitochondrial outer membrane potential

For the flow cytometric evaluation of mitochondrial membrane potential (Δψ_m_), cells were stained with the fluorescent lipophilic cationic dye JC‐1 (5,5′,6,6′‐tetrachloro‐1,1′,3,3′‐tetraethyl‐imidacarbocyanine iodide; Sigma‐Aldrich). JC‐1 possesses the unique ability to differentially label mitochondria with low and high membrane potential (Δψ_m_). In mitochondria with high (Δψ_m_), JC‐1 forms multimeric aggregates that emit orange–red light (wavelength of 590 nm) when excited at 488 nm. In mitochondria with low (Δψ_m_), JC‐1 forms monomers that emit green light (525–530 nm) when excited at 488 nm. For labelling with JC‐1, cells were collected and incubated for 20 min with 1 μg·mL^−1^ of JC‐1 in culture medium at 37 °C in the dark. Cells were centrifuged to remove the supernatant. Cell pellets were suspended in PBS, and the ratio of red/green fluorescence intensity was determined by flow cytometry. For each sample, 10 000 events were recorded at a flow rate of 200–300 cells·s^−1^, and debris and aggregates were gated out by establishing a region around the population of interest in the forward scatter/side scatter dot plot on a log scale. Compensation between FL1 and FL2 was carefully adjusted according to the manufacturer's instructions. Green and orange–red fluorescences were measured in FL‐1 and FL‐2 channel, respectively. The samples were analysed on CyAn‐ADP flow cytometer (Dako). Data acquisition was performed using Summit software (Dako).

## 3 Results

### 3.1 *MET* oncogene‐addicted carcinoma cells are susceptible to HSP27 suppression

**Figure 1:**
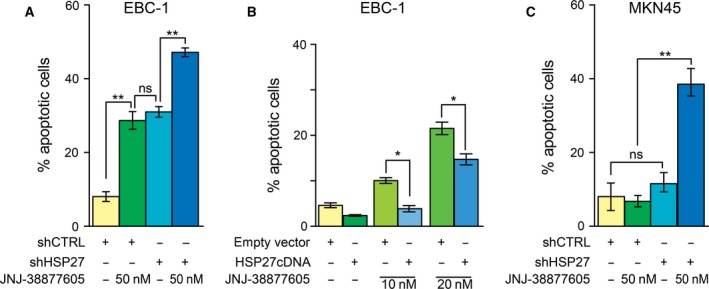
Protection from apoptosis of MET‐addicted cancer cell lines by HSP27. The indicated cell lines were transduced to express either the shHSP27 or control scrambled sh (shCTRL) (A,C) or either the HSP27 cDNA or the corresponding empty vector (B). Silenced cells were examined 72 h after transduction. (A) The HSP27‐silenced EBC‐1 lung cancer cells were treated with the MET inhibitor JNJ‐38877605 for further 48 h at the indicated concentrations; (B) HSP27‐overexpressing EBC‐1 cells were treated with the MET inhibitor JNJ‐38877605 for further 48 h at the indicated concentrations; (C) the HSP27‐silenced MKN45 gastric cancer cells were treated with the MET inhibitor JNJ‐38877605 for further 48 h at the indicated concentrations. Apoptotic cells were measured using FACS analysis of AnnV and DAPI staining. Significance was calculated using the one‐way ANOVA performed using graphpad prism (GraphPad Software, San Diego, CA, USA): *P < 0.05; **P < 0.001.

HSP27 silencing was alone able to commit the EBC‐1 lung carcinoma cells to death (Figs 1A and S1A). These cells display *MET* gene amplification and are addicted to the *MET* oncogene activation as shown by the induction of cell death by the selective MET kinase inhibitor JNJ‐38877605 (Fig. 1A). Cell death was further increased when the MET inhibitor was administered to HSP27‐silenced cells (Fig. 1A). In line, HSP27 overexpression (Fig. S1B) protected EBC‐1 cells from JNJ‐38877605 (Fig. 1B).

The MKN45 cells are known as MET‐addicted cells as inhibition of this kinase resulted in cell cycle arrest (Bertotti *et al*., 2009; Musiani *et al*., 2014). These cells were not affected by HSP27 silencing alone (Figs 1C and S1C). However, in HSP27‐silenced cells the MET inhibitor JNJ‐38877605 induced cell apoptotic death (Fig. 1C), although HSP27 expression is increased by the treatment with the inhibitor (Musiani *et al*., 2014). Altogether, these data show that in MET‐addicted carcinoma cell lines, HSP27 silencing affected cell survival and allowed MET inhibitor to exert a full cytotoxic effect.

### 3.2 EGFR‐addicted carcinoma cells are susceptible to HSP27 suppression

**Figure 2:**
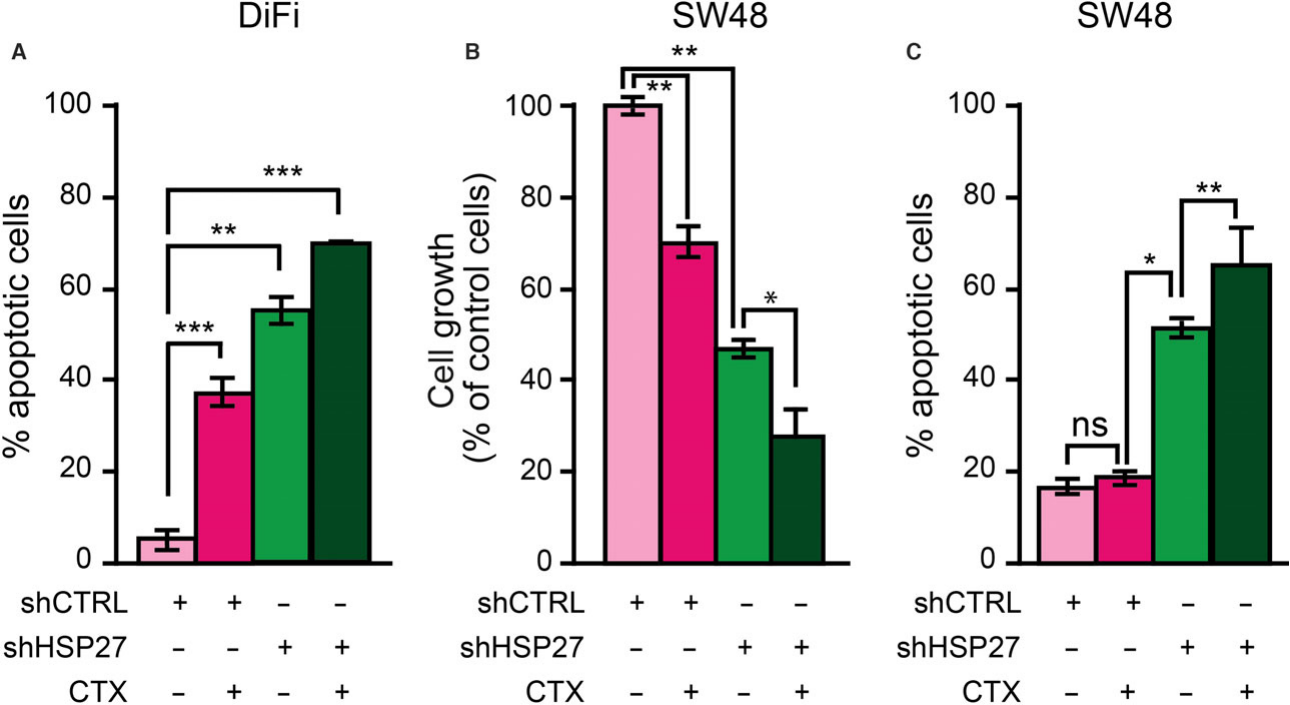
Protection from apoptosis of EGFR‐addicted cancer cell lines by HSP27. The indicated CRC cell lines were transduced to express either the shHSP27 or control scrambled sh (shCTRL). Silenced cells were examined 72 h after transduction. (A) DiFi cells were treated with the EGFR antibody cetuximab (CTX, 5 nm for further 48 h); apoptotic cells were measured using FACS analysis of AnnV and DAPI staining. (B) SW‐48 cells were treated with the EGFR antibody cetuximab (CTX) at 5 nm for further 48 h, and cell growth was evaluated with the CellTiter‐Glo assay; (C) SW‐48 cells were treated with the EGFR antibody cetuximab (CTX) at 5 nm for further 48 h, and apoptotic cells were measured as in (A). Significance was calculated using the one‐way ANOVA performed using graphpad prism: *P < 0.05; **P < 0.001; ***P < 0.0005.

HSP27 silencing was alone able to induce the apoptotic death of the CRC‐derived DiFi cells (Figs 2A and S2A), which are addicted to EGFR because of receptor amplification. These cells were susceptible to anti‐EGFR antibody cetuximab (CTX), which was alone able not only to block proliferation (Medico *et al*., 2015; Musiani *et al*., 2014) but also to affect cell survival (Fig. 2A). HSP27 silencing further increased the susceptibility of DiFi cells to CTX (Fig. 2A).

The CRC‐derived SW48 cells are also classified as EGFR‐addicted because CTX was alone able to block cell proliferation (Musiani *et al*., 2014; Troiani *et al*., 2014), as also shown in Fig. 2B. However, CTX was not able to drive cells to death (Fig. 2C). HSP27 silencing (Fig. S2B) alone affected the survival of SW48 cells and sensitized them to the EGFR inhibitor so that the combined treatments resulted in increased cell death (Fig. 2C), although HSP27 expression is upregulated by the treatment with the inhibitor (Musiani *et al*., 2014). Interestingly, no correlation (Fig. S3) was found between the addiction to cetuximab and basal level of expression of HSP27 in 150 colorectal cancer cell lines treated with the drug, as reported by Medico *et al*. (2015). Altogether, these data show that in EGFR‐addicted carcinoma cell lines, HSP27 silencing affected cell survival and allowed the EGFR inhibitor to exert a full cytotoxic effect.

### 3.3 BRAF‐addicted carcinoma cells are susceptible to HSP27 suppression

**Figure 3:**
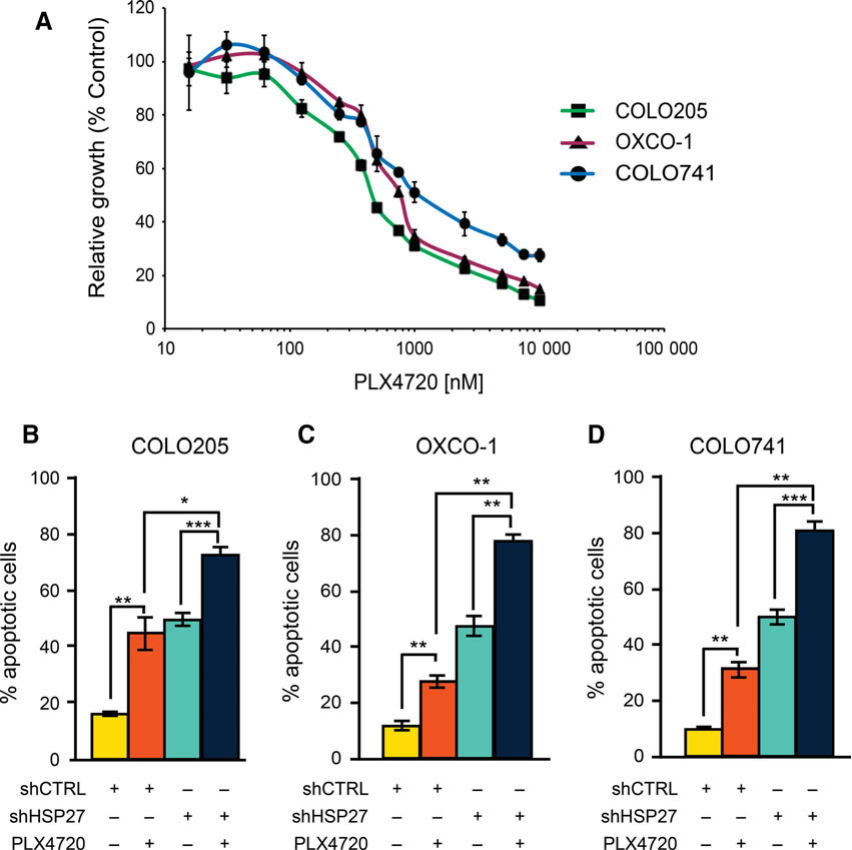
Protection from apoptosis of BRAF‐addicted cancer cell lines by HSP27. (A) Proliferation of the CRC COLO205 and OXCO‐1 cell lines and of the melanoma COLO741 cell line, evaluated using CellTiter‐Glo assay after cell plate treatment for 48 h with the indicated concentrations of the BRAF inhibitor PLX4720. (B–D) The indicated cell lines were transduced to express either the shHSP27 or control scrambled sh (shCTRL). Apoptosis of the same cell lines 72 h after cell transduction and treated, where indicated, with PLX4720 (1 μm) for further 48 h. Apoptotic cells were measured using FACS analysis of AnnV and DAPI staining. Significance was calculated using the one‐way ANOVA performed using graphpad prism: *P < 0.05; **P < 0.001; ***P < 0.0005.

The BRAF‐mutated COLO205 and OXCO‐1 CRC cells and the COLO741 melanoma cells display impaired proliferation when treated with BRAF inhibitor vemurafenib (Prahallad *et al*., 2012). Figure 3A shows that the BRAF inhibitor PLX4720, the structurally closely related precursor of vemurafenib, similarly affected the proliferation of these cell lines. Also the survival of these cell lines was impaired by the BRAF inhibitor (Fig. 3B–D). In all three cell lines, HSP27 knockdown (Fig. S4) resulted in some degree of apoptotic death, which was achieved fully after the treatment of silenced cells with the BRAF inhibitor (Fig. 3B–D), although BRAF inhibitor treatment induced an increase in the HSP27 expression (Fig. S4). Altogether, these data show that the BRAF‐addicted cells are susceptible to HSP27 silencing and fully committed to death by the combined HSP27 silencing and BRAF inhibition.

### 3.4 RAS‐addicted cells are susceptible to HSP27 suppression

**Figure 4:**
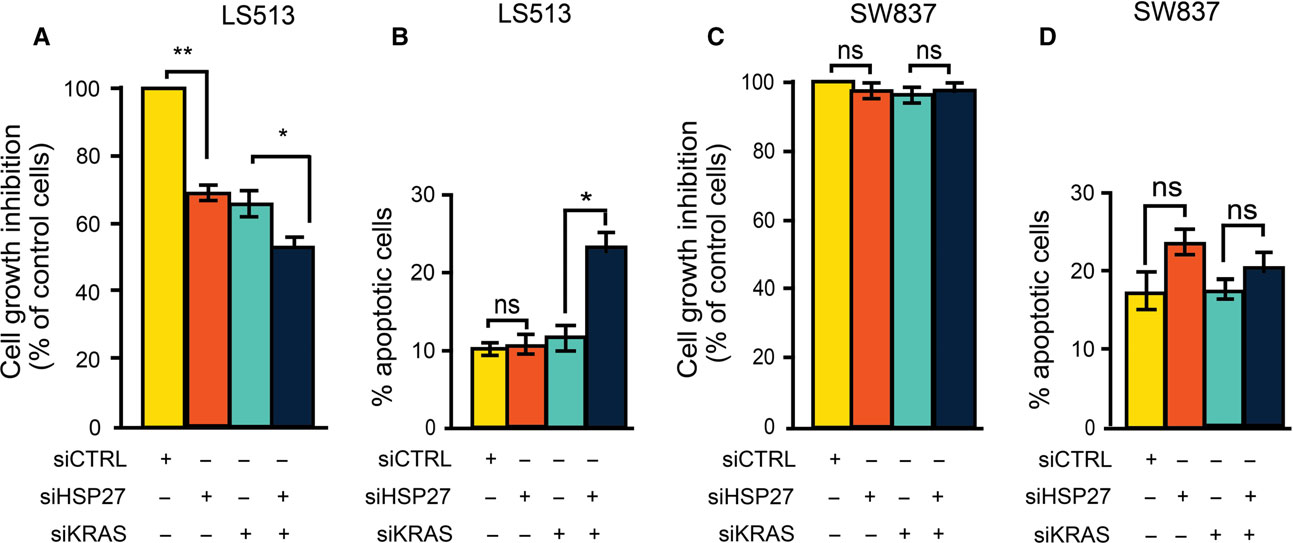
Protection from apoptosis of CRC cell lines addicted to KRAS by HSP27. Where indicated, either KRAS or HSP27 was silenced using small interfering RNA pools. Nontargeting pool was expressed as control (siCTRL). (A) LS513 cells were transfected to express the indicated siRNA for 72 h, and cell growth was evaluated using trypan blue exclusion assay. (B) The same cells were transfected as in (A), and apoptosis was measured using FACS analysis of AnnV and DAPI staining. (C) The SW837 cells were transfected and cell growth was evaluated as in (A). (D) The same cells were transfected and apoptosis measured as in (B). Significance was calculated using the one‐way ANOVA performed using graphpad prism: *P < 0.05; **P < 0.001; ns: not significant.

HSP27 expression was knocked down in two CRC cell lines (LS513 and SW837; Fig. S5), carrying two different KRAS mutations (G12D and G12C, respectively). These cell lines were previously described (Lamba *et al*., 2014) as either addicted (LS513) or not addicted (SW837) to the KRAS‐activated pathway. In LS513 cells, either KRAS or HSP27 suppression reduced cell proliferation (Fig. 4A), but neither was alone able to commit cells to death (Fig. 4B). Conversely, the combination further reduced cell proliferation (Fig. 4A) and was effective in driving cell to death (Fig. 4B). The SW837 cells were almost unaffected by KRAS suppression and also by the combination of KRAS and HSP27 silencing (Fig. 4C,D).

### 3.5 HSP27 suppression results in the modulation of apoptosis‐associated proteins and mitochondrial priming

As it has been reported that HSP27 might protect cells by binding proteins of the BCL2 family, first we studied the proteins of this family with either pro‐ or anti‐apoptotic function in the MET‐addicted MKN45 cells after HSP27 stable silencing. These cells were studied first because, as described above (Fig. 1), MKN45 cells (i) were not affected by HSP27 silencing alone; (ii) did not die but no longer proliferated when treated with MET inhibitor; and (iii) were driven to apoptosis when the MET inhibitor was added to HSP27‐silenced cells.

**Figure 5:**
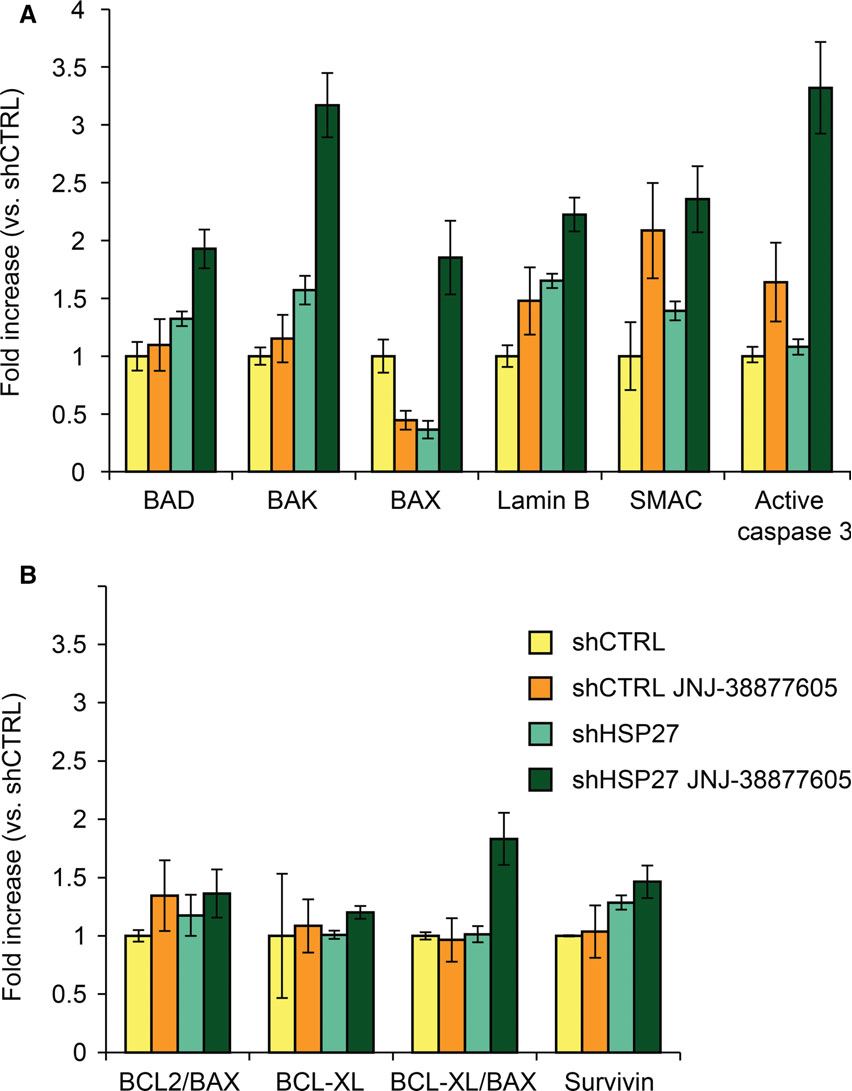
Evaluation of the expression of apoptosis‐related proteins in MKN45 cells where HSP27 was silenced as in panel (C) of Fig. 1 versus the expression in cell transduced with shCTRL. Cells were treated with JNJ‐38877605 (50 nm for 36 h) where indicated. (A) Pro‐apoptotic proteins and (B) anti‐apoptotic proteins were measured using Bio‐Plex assay.

We previously showed (Musiani *et al*., 2014) that the pro‐apoptotic BIM protein increased when MET‐addicted cells were treated with the MET inhibitor but was not affected by HSP27 silencing; conversely, the anti‐apoptotic MCL1 protein decreased as a result of the combination of HSP27 silencing and MET inhibition. Further screening of anti‐BCL2 family proteins with the antibodies of the Bio‐Plex assay showed (Fig. 5A,B) that MET inhibition in HSP27‐silenced MKN45 cells also resulted in the increase in the BAK and BAX proteins and in active caspase‐3 and lamin B. Conversely, in these cells, where HSP27 silencing alone did not result in cell death, accordingly pro‐ and anti‐apoptotic proteins were almost unaffected by the HSP27 silencing alone.

Pro‐ or anti‐apoptotic proteins were then analysed using the Bio‐Plex assay in the BRAF‐addicted OXCO‐1, COLO741 and COLO205 cells. Figure S6A shows the high basal level of BAX and BAK in OXCO‐1 and COLO741 cells, respectively, in line with their propensity to death. The effect of HSP27 silencing onto pro‐ and anti‐apoptotic proteins in these cell lines, which undergo dramatic apoptosis when silenced (Fig. 3B,C), was barely detected because of massive cell death. A modest increase in BAK was detectable in the COLO205 cells mostly after the treatment with the targeted agent (Fig. S6B), and only the increase in active caspase 3 was measured in all cell lines (Fig. S6C).

**Figure 6:**
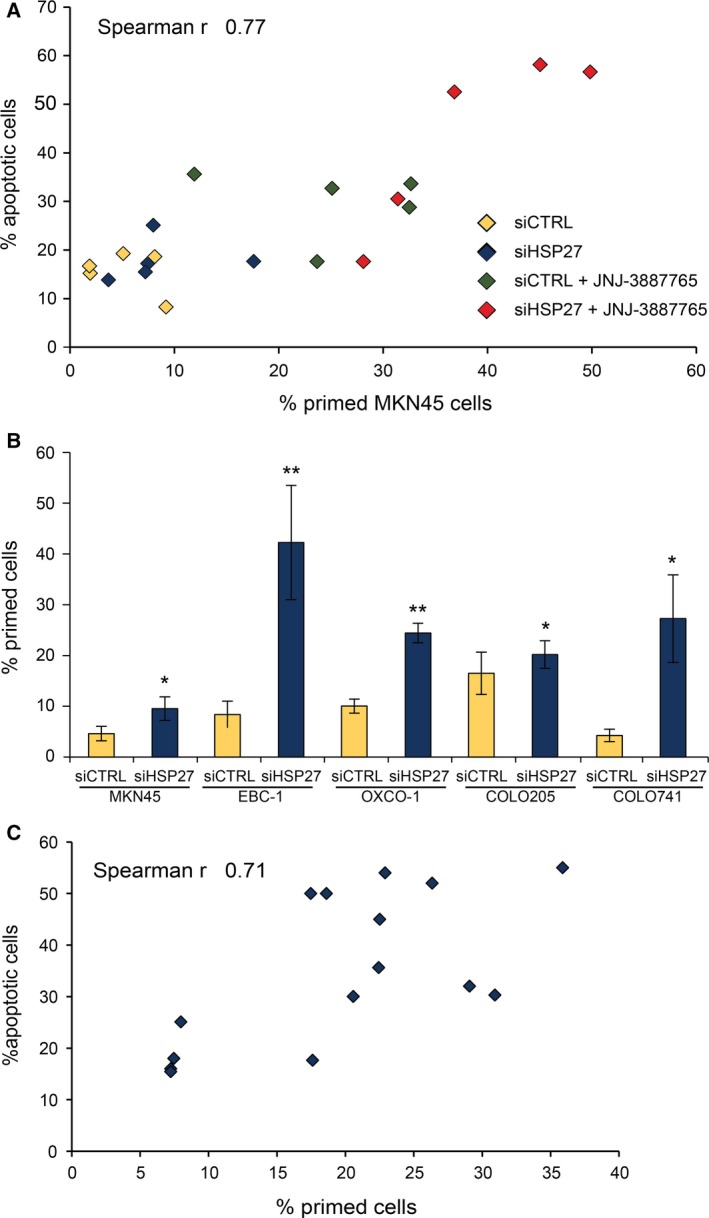
Mitochondrial priming of oncogene‐addicted cancer cell lines was correlated with propensity to apoptotic death induced by HSP27 silencing. The percentage of cells showing mitochondrial priming to death, that is increased MOMP, was calculated by measuring accumulation of JC‐1 green monomers with FACS analysis. Where indicated, cells were transfected with either HSP27‐specific (siHSP27) or control small interfering RNA (siCTRL). (A) Plot showing the correlation in treated MKN45 cells between the percentage of cells showing MOMP increase (primed) and that of cells showing annexin V and DAPI staining (apoptotic), measured with FACS analysis. The MET inhibitor JNJ‐605 was added 72 h after transfection, and cells were examined after 48 h. (B) Basal level of priming and levels achieved after HSP27 suppression in the indicated cell lines. Significance was calculated using the one‐way ANOVA performed using graphpad prism: *P < 0.05; **P < 0.001. (C) Plot showing the correlation between level of cell priming and readiness to apoptosis in cells where HSP27 was silenced. Each diamond represents one experiment in which priming and apoptosis were measured in the same cells. In all the cell lines of panel (B), the percentage of cells showing MOMP increase (primed) and that of cells showing annexin V and DAPI staining (apoptotic) after HSP27 silencing were measured in at least three separate experiments.

As the net balance of pro‐ and anti‐apoptotic proteins results in the formation of mitochondrial pores and possibly in the consequent decrease in mitochondrial outer membrane permeabilization (MOMP), we measured MOMP using the cationic dye JC‐1. JC‐1 exhibits potential‐dependent accumulation in mitochondria, indicated by fluorescence emission shift from red to green, and can be quantified using flow cytometry (Szilágyi *et al*., 2006). MOMP decrease results in proximity of cells to apoptosis threshold, a phenomenon called priming. Figure 6A shows that in MKN45 cells, in line with data of Fig. 1, there is a correspondence between priming and cell death. HSP27 silencing and the MET inhibitor alone did not change priming and survival (green and blue diamonds), while the combined treatment with HSP27 siRNAs and MET inhibitor resulted in increased priming and switching the effect of the MET inhibitor towards cell death (red diamonds).

Then, we measured priming of all the other studied cells and found that a basal level of priming in COLO205 cells and to a lesser extent in OXCO‐1 cells (Fig. 6B) in line with their propensity to respond to the targeted agent anti‐BRAF drug (Fig. 3). In all but MKN45 cells, HSP27 silencing alone increased priming to death (Fig. 6B). Conversely, the treatment of cells with control siRNA and the relevant targeted agent resulted in a modest increase in mitochondrial priming (Fig. S7).

As different level of basal and HSP27 silencing‐induced priming appeared correlated with the effects on cell death shown in Figs 1, 2, 3, we measured in parallel priming and cell death in the same cell lines following silencing of HSP27 with siRNAs. As shown in Fig. 6C, we found a correlation between cell death and priming caused by HSP27 silencing.

## 4 Discussion

This study shows that HSP27 protects oncogene‐addicted cancer cells from the activation of the mitochondrial pathway of apoptosis, measured as increased MOMP and increased levels of pro‐apoptotic effector proteins. These parameters define mitochondrial readiness to trigger apoptosis, the so‐called mitochondrial priming to death. This pathway is likely to be often intrinsically active in oncogene‐addicted cells, likely because of oncogene overactivation. Moreover, targeted agents can activate the same pathway, and we show here that HSP27 silencing made cells more susceptible to these agents. The protective role of HSP27 makes cells surviving, and the net effect might be the interference of HSP27 with targeted therapies. This is important as HSP27 expression is increased in several cancer histotypes (for a review, see Garrido *et al*., 2006). Moreover, we had shown previously that HSP27 expression is increased by cell treatment with targeted agents through the activation of the HSF1 and HIF1α pathway (Musiani *et al*., 2014).

We show that cancer cells more susceptible to targeted therapy, such as the colorectal BRAF‐addicted COLO205, which undergo cell death when treated with the specific targeted agent, might even show a basal level of mitochondrial priming, as well as cancer cells more susceptible to chemotherapy (Ni Chonghaile *et al*., 2011), and are consistently susceptible to the loss of HSP27 which causes increased MOMP. MOMP has been considered a point of no return in the apoptotic programme for a long time. Conversely, our data as well as other authors’ data (for a review, see Tait and Green, 2010) show that cancer cells propagated in culture can resist MOMP. It has been proposed that the ability of cells to survive chronic MOMP depends on a few mitochondria that evade permeabilization and repopulate the cell (Tait *et al*., 2010). Moreover, it has been recently also shown that a limited level of MOMP might be not only present and compatible with life of cancer cells, but could even contribute in promoting oncogenesis by enabling DNA damage (Ichim *et al*., 2015; Liu *et al*., 2015).

MOMP and mitochondrial priming depends on the balance between anti‐ and pro‐apoptotic proteins of the BCL2 family (Certo *et al*., 2006; Chipuk *et al*., 2010), which are expressed at different level in different tissues and cell types (Placzek *et al*., 2010). Among these proteins, the effector proteins BAX and BAK undergo oligomerization when activated, form pores in the mitochondrial outer membrane and finally cause apoptosis (Kluck *et al*., 1997; Wei *et al*., 2001). The effector BAK and BAX can be activated by the BH3‐only proteins BIM and BID, also known as activators (Sarosiek *et al*., 2013). Both effectors and activators can be inhibited by the anti‐apoptotic members of the family (Certo *et al*., 2006). Induction of apoptosis through BCL2 protein is a critical component of effective targeted therapies (Hata *et al*., 2015). It has been previously demonstrated that in particular, BIM increase is invariably associated with the targeting of different oncogenic kinases that results in the down‐modulation of MEK–ERK and PI3K–AKT pathways (for a review, see Hata *et al*., 2015). We already reported BIM increase in response to the MET tyrosine kinase inhibitor and a decreased level of the anti‐apoptotic MCL1 after the silencing of HSP27 (Musiani *et al*., 2014). This is in line with the report that MCL1 down‐modulation is required for the full activity of targeted inhibitors of the pathways activated by oncogenic tyrosine kinase receptors, for example by those of HER family (Faber *et al*., 2009; Misale *et al*., 2015). We show here that MET inhibition in HSP27‐silenced cells was also associated with the increase in BAK and BAX proteins, whose increased levels have been correlated with better responses of cancer cells to chemotherapy (Lange *et al*., 2003; Lindner *et al*., 2013; Luo *et al*., 2015). These data confirm that drugs targeting BCL2 proteins are promising allies of targeted agents to eradicate tumours (for a review, see Hata *et al*., 2015).

Silencing of HSP27 turned the antiproliferative effect of targeted inhibitors into full cytotoxic activity. Experiments demonstrated that HSP27 loss weakened the anti‐apoptotic machinery of the MET‐addicted cells. As mentioned above, not only BAX and BAK proteins increased, but also the pro‐apoptotic BIM increased and the anti‐apoptotic MCL1 decreased (Musiani *et al*., 2014). This implies that the combination of HSP27 silencing and MET kinase inhibition could more steadily control the growth of MET‐addicted tumours and suggests that a similar mechanism drives full cytotoxicity driven by HSP27 silencing and targeted agents.

HSP27 might protect cells from mitochondrial apoptosis at several levels (for a review, see Acunzo *et al*., 2012). For example, numerous studies report that the caspase cascade inactivation is a consequence of HSP27 binding with caspase‐3 and cytochrome *c* release from the mitochondria. Here, we show however that the protective effect might occur also earlier in the mitochondrial pathway of apoptosis, by blocking mitochondrial permeabilization. This could be due to the known ability of HSP27 to stabilize directly or indirectly upstream molecules such as AKT and BAX (Arrigo, 2007; Havasi *et al*., 2008; Zhang *et al*., 2015).

## 5 Conclusions

The protective role of HSP27 makes cells surviving, and the net effect might be the interference of HSP27 with targeted therapies. Thus, agents targeting HSP27 such as OGX‐427 (Baylot *et al*., 2011; Matsui *et al*., 2009), which is already undergoing clinical trials, and aptamers (Gibert *et al*., 2011), could be envisaged as a therapeutic approach to sensitize cells to targeted agents.

## Author contributions

MFD and MO conceived and designed the project; JDK, DM, MO and SL acquired and analysed the data; MFD, MO and DM interpreted the data; and JDK and MFD wrote the manuscript.

## Supporting information


**Fig. S1.** HSP27 expression in MET‐addicted cancer cell lines.

**Fig. S2.** HSP27 expression in EGFR‐addicted cancer cell lines.

**Fig. S3.** Correlation between HSP27 expression and cell response to a targeted drug.

**Fig. S4.** HSP27 expression in BRAF‐addicted cancer cell lines.

**Fig. S5.** HSP27 expression in KRAS‐expressing cancer cell lines.

**Fig. S6.** Evaluation of apoptosis‐related proteins in RAF‐addicted cancer cell lines, measured using Bio‐Plex assay (A) Basal level of expression; (B) measure of BAK and of active caspase 3 (C) in cells where HSP27 was silenced as in panels B–D of Fig. 3 versus the expression in cell transduced with shCTRL. Cells were treated with PLX4720 (1 μm for 48 h) where indicated. Significance was calculated using Student's t‐test: *P = 0.05; **P < 0.05; ***P < 0.005.

**Fig. S7.** Mitochondrial priming achieved after treatment of the indicated cell lines with the relevant drug to which they are addicted.
