# Supplementary figures and images for: Heat‐shock protein 27 (HSP27, HSPB1) is synthetic lethal to cells with oncogenic activation of MET, EGFR and BRAF
Source: Mol Oncol. 2017 May 8;11(6):599–611. doi: 10.1002/1878-0261.12042 (PMC5467498; doi:10.1002/1878-0261.12042)

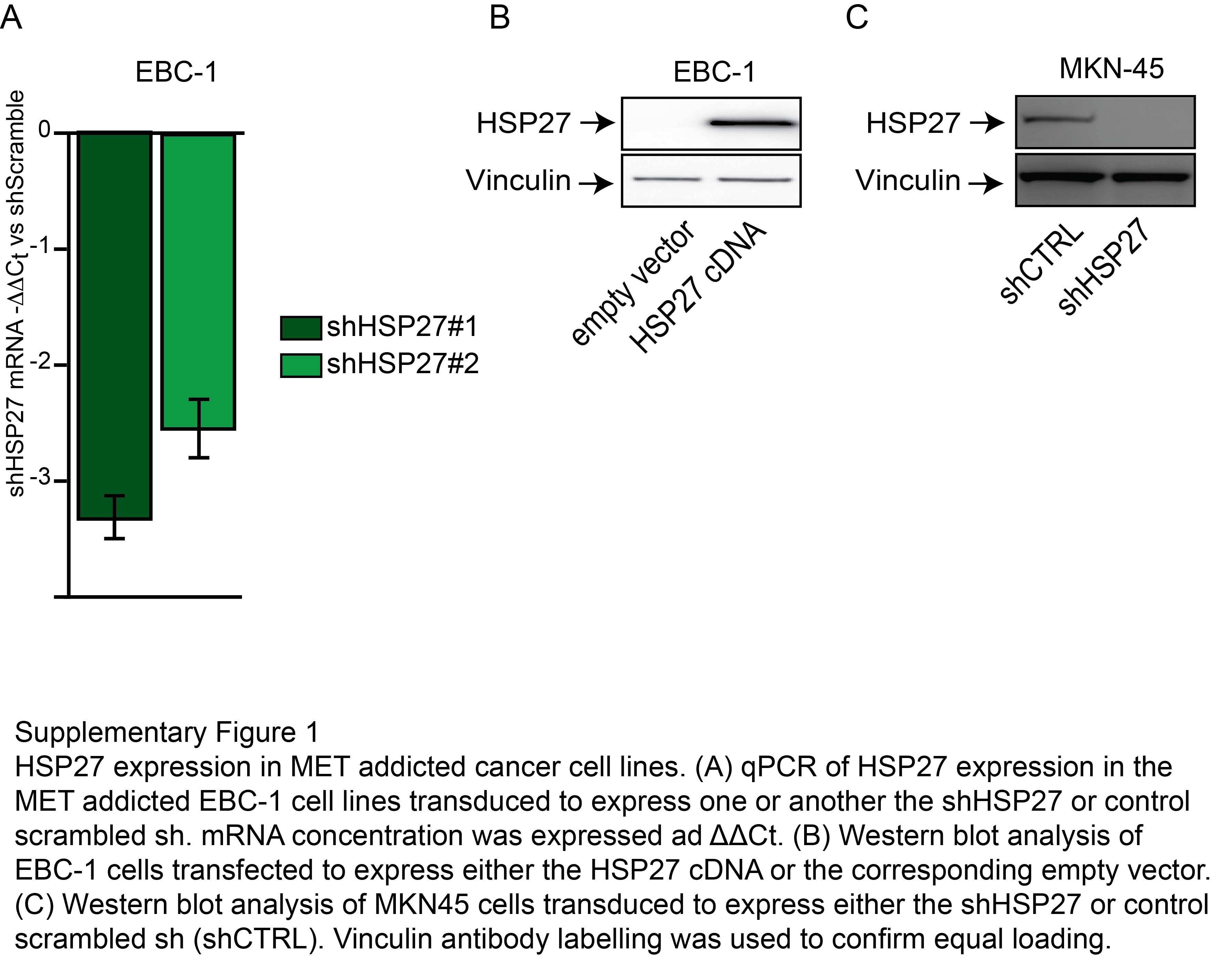

Supplement: Supplementary file 1 — Fig. S1. HSP27 expression in MET‐addicted cancer cell lines. [file MOL2-11-599-s001.jpg]

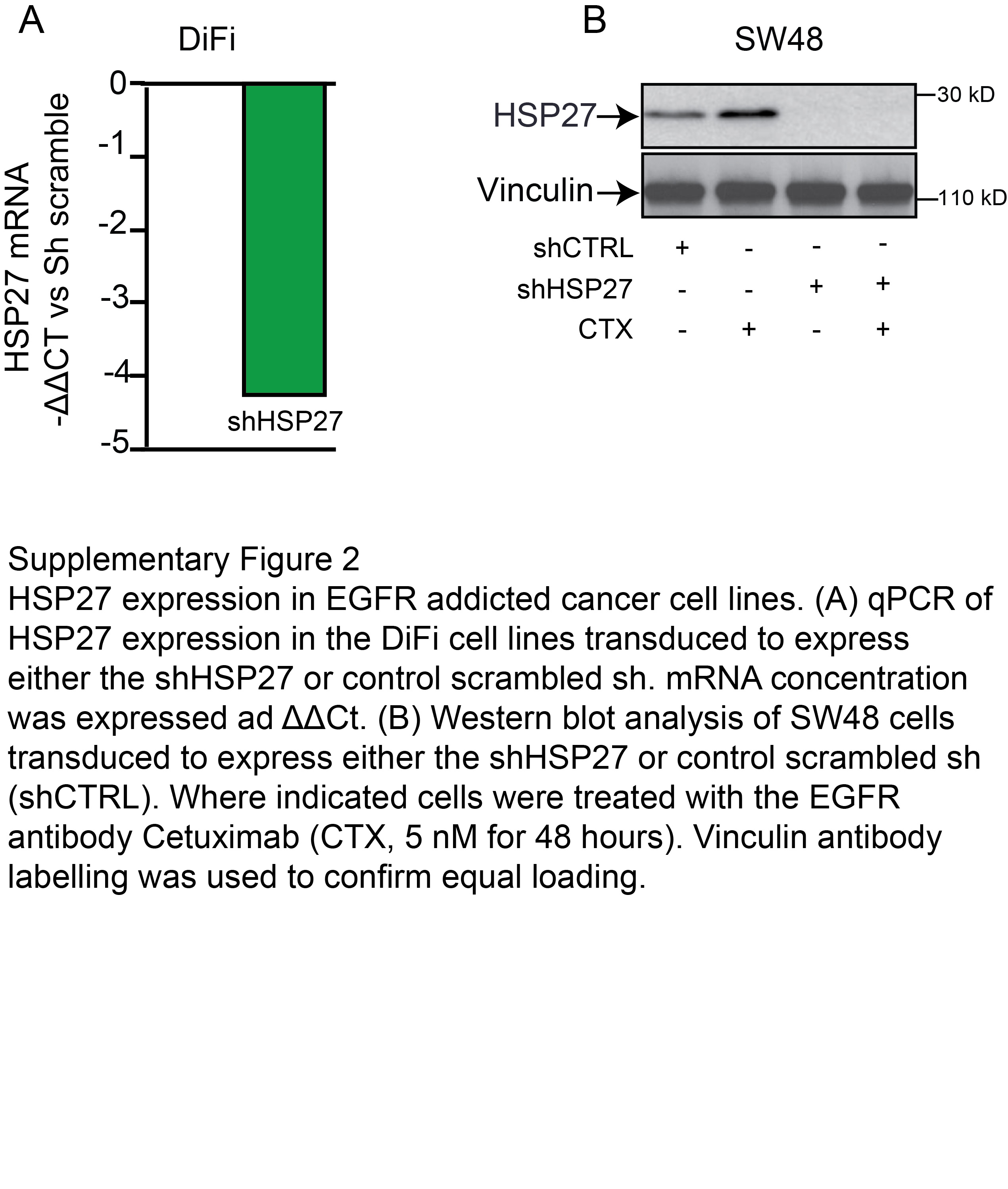

Supplement: Supplementary file 2 — Fig. S2. HSP27 expression in EGFR‐addicted cancer cell lines. [file MOL2-11-599-s002.jpg]

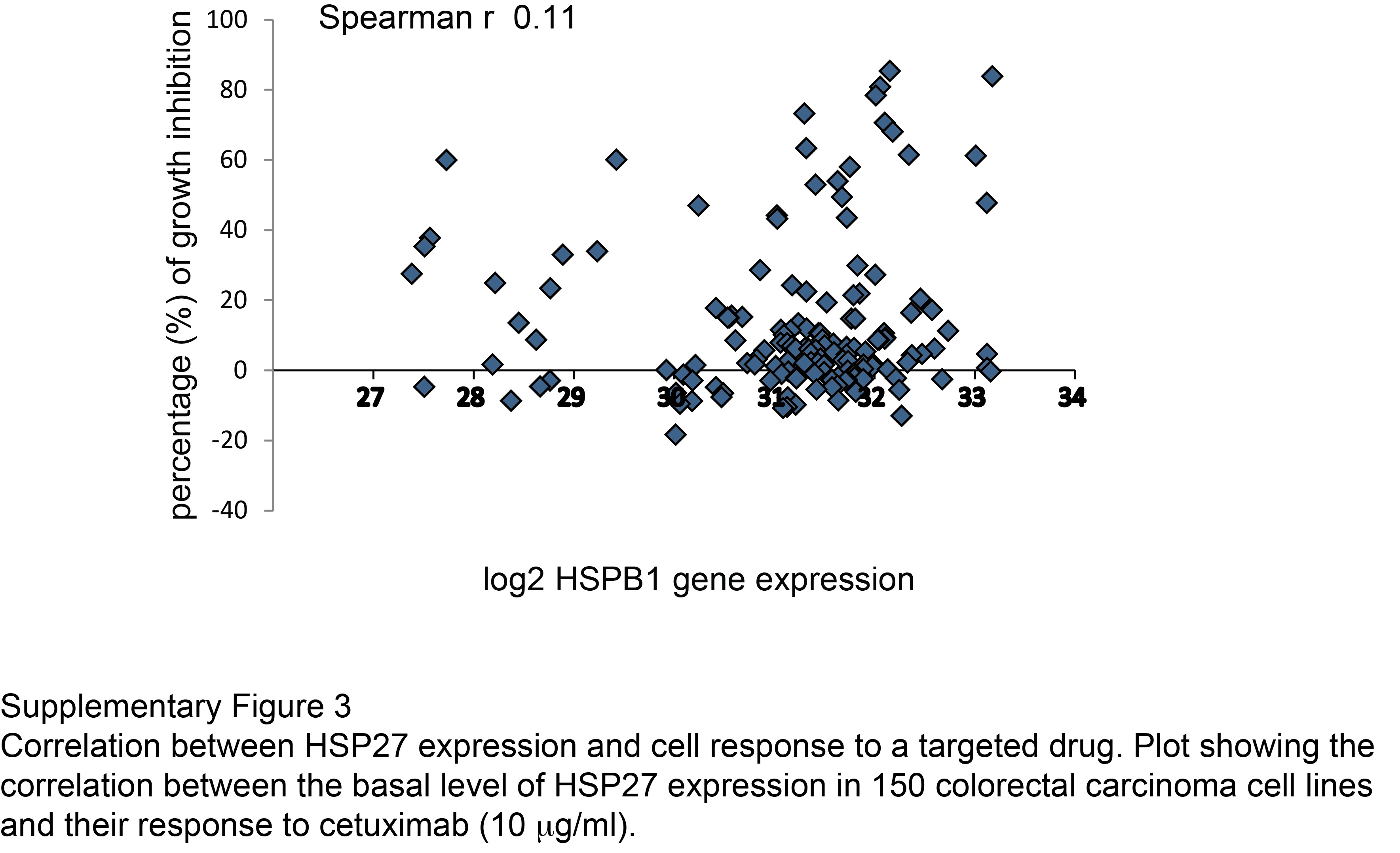

Supplement: Supplementary file 3 — Fig. S3. Correlation between HSP27 expression and cell response to a targeted drug. [file MOL2-11-599-s003.jpg]

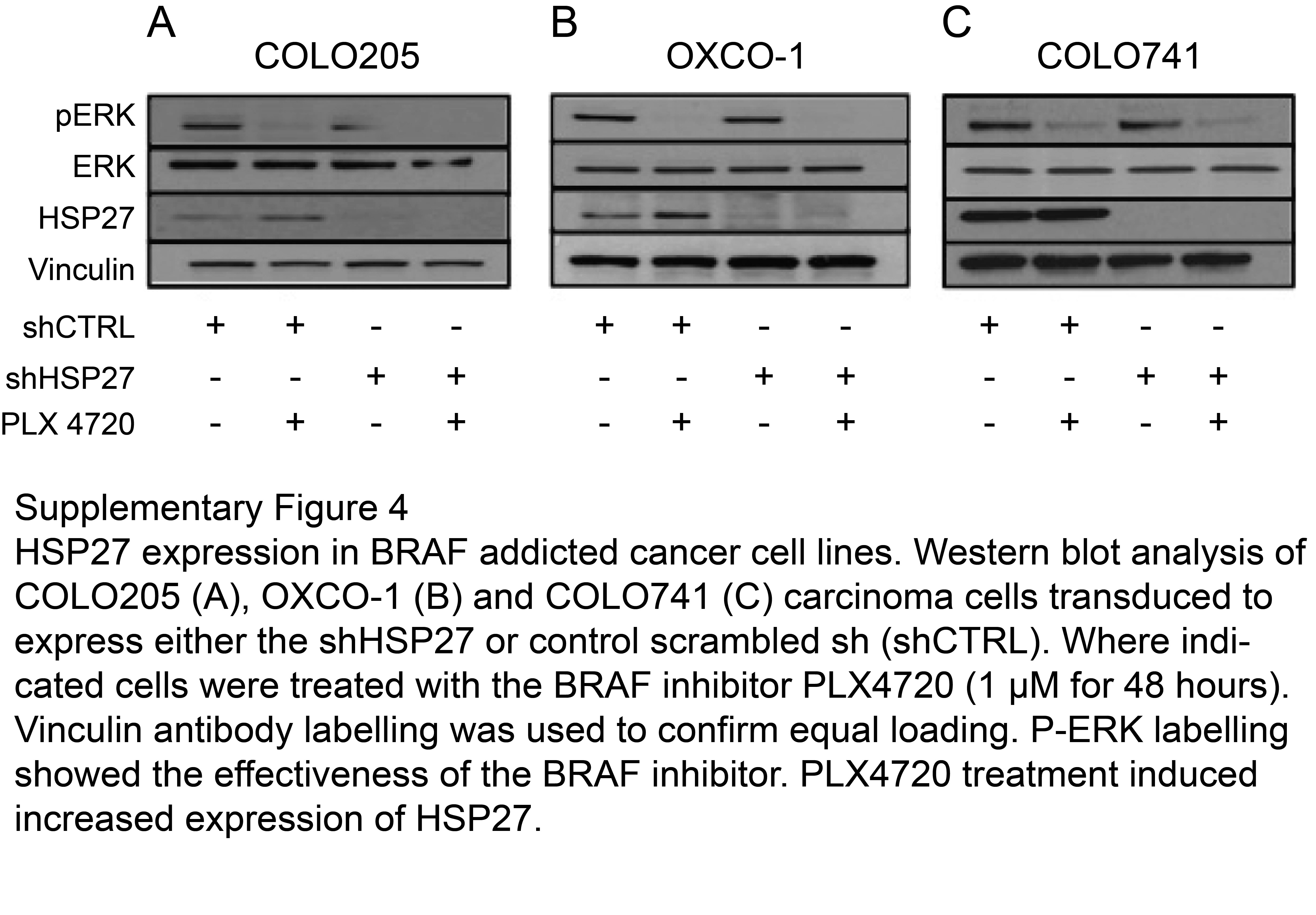

Supplement: Supplementary file 4 — Fig. S4. HSP27 expression in BRAF‐addicted cancer cell lines. [file MOL2-11-599-s004.jpg]

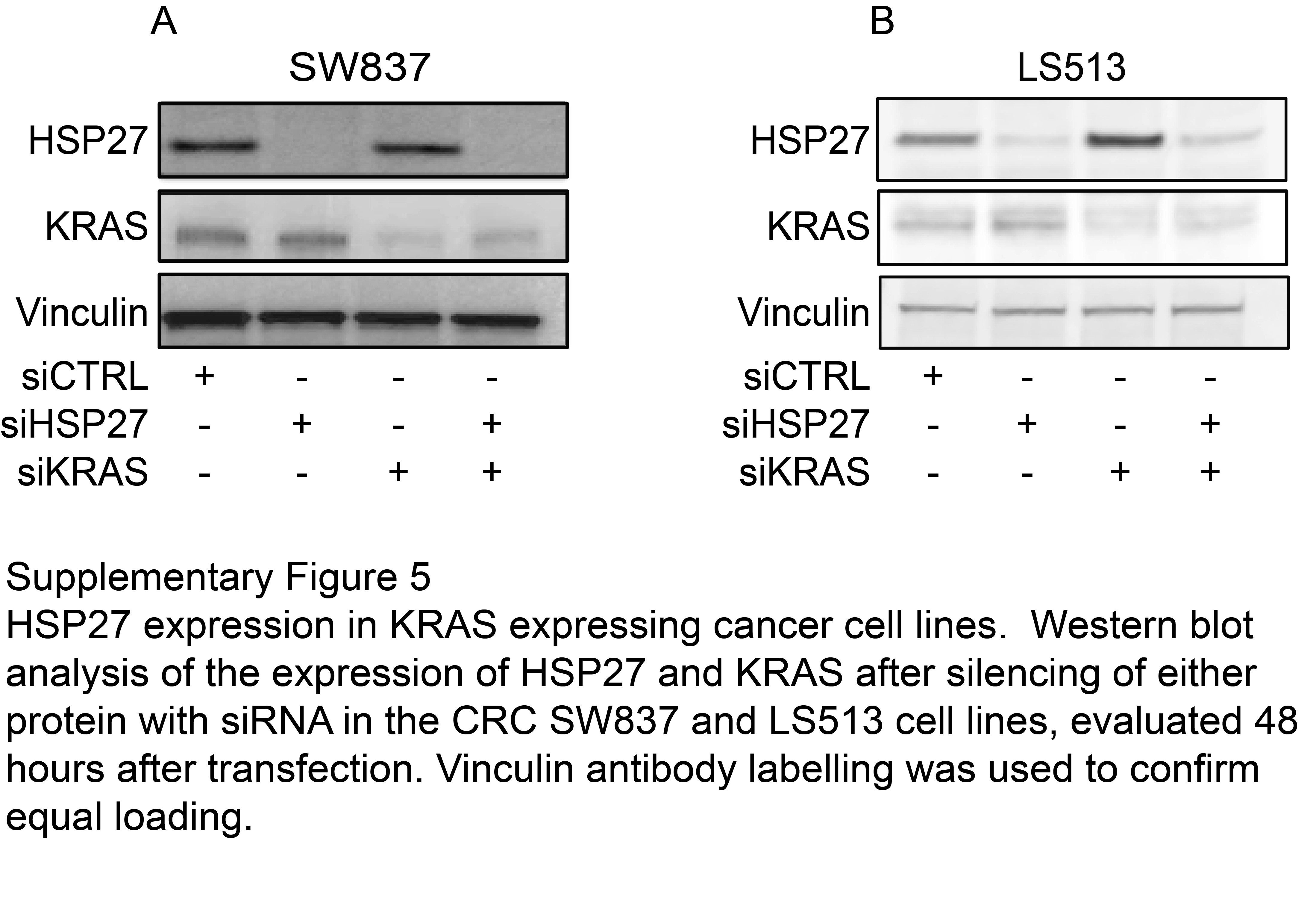

Supplement: Supplementary file 5 — Fig. S5. HSP27 expression in KRAS‐expressing cancer cell lines. [file MOL2-11-599-s005.jpg]

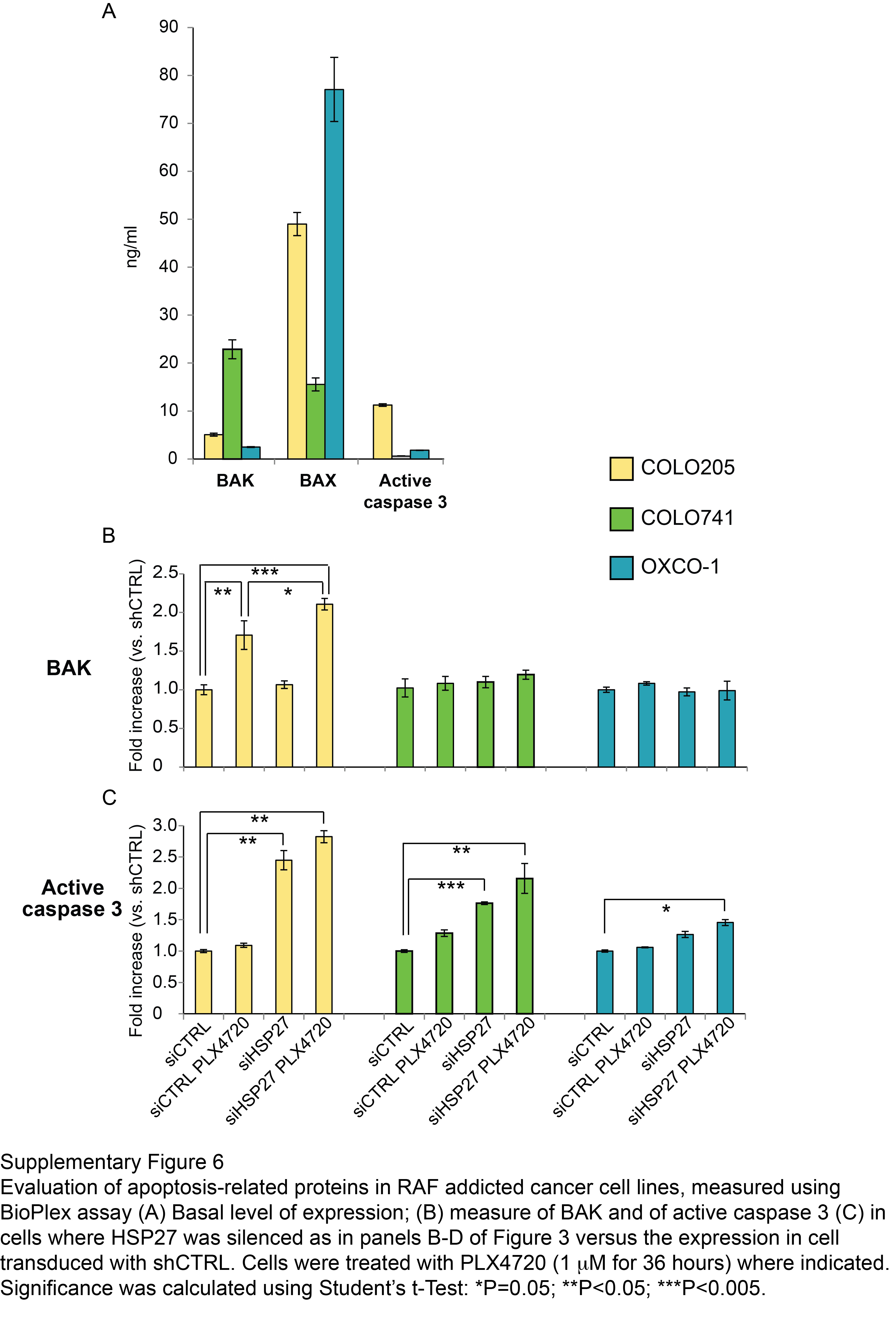

Supplement: Supplementary file 6 — Fig. S6. Evaluation of apoptosis‐related proteins in RAF‐addicted cancer cell lines, measured using Bio‐Plex assay (A) Basal level of expression; (B) measure of BAK and of active caspase 3 (C) in cells where HSP27 was silenced as in panels B–D of Fig. 3 versus the expression in cell transduced with shCTRL. Cells were treated with PLX4720 (1 μm for 48 h) where indicated. Significance was calculated using Student's t‐test: *P = 0.05; **P < 0.05; ***P < 0.005. [file MOL2-11-599-s006.jpg]

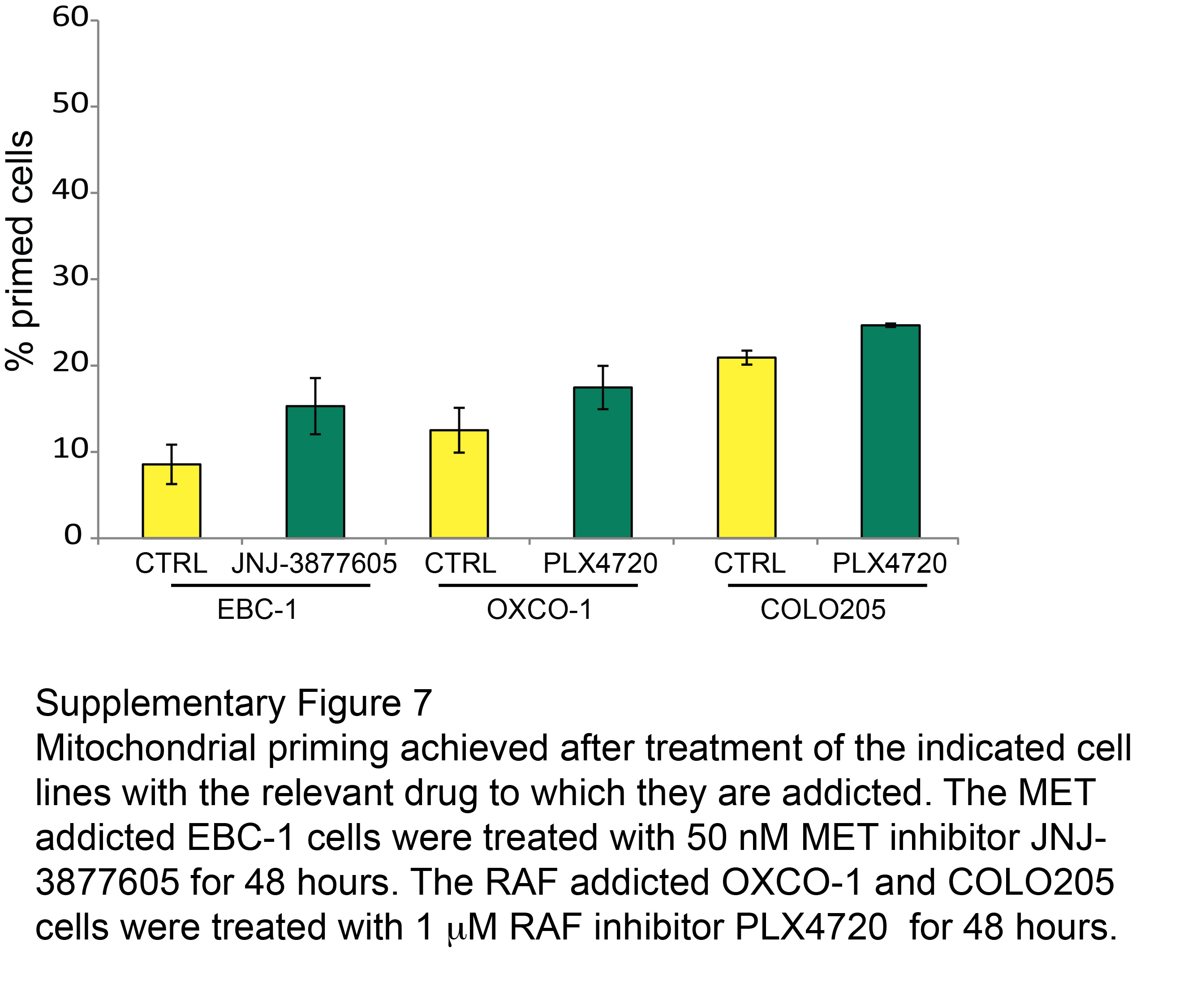

Supplement: Supplementary file 7 — Fig. S7. Mitochondrial priming achieved after treatment of the indicated cell lines with the relevant drug to which they are addicted. [file MOL2-11-599-s007.jpg]
